# A Review of Local Consent Practices for Colorectal Surgery With a Focus on Postoperative Bowel Dysfunction

**DOI:** 10.7759/cureus.86285

**Published:** 2025-06-18

**Authors:** Mahir H Sampat, Sanaa Elgaddal

**Affiliations:** 1 General Surgery, The Royal Wolverhampton National Health Service (NHS) Trust, Wolverhampton, GBR; 2 Colorectal Surgery, The Royal Wolverhampton National Health Service (NHS) Trust, Wolverhampton, GBR; 3 General and Colorectal Surgery, New Cross Hospital, Wolverhampton, GBR

**Keywords:** lower gastrointestinal or colorectal surgery, quality improvement and patient safety, sphincter-sparing oncologic surgery, surgical consent, surgical informed consent

## Abstract

Aim: Long-term bowel dysfunction is a significant consequence of colorectal surgery, having a major impact on quality of life. Despite this, patients often do not receive adequate information while consenting to surgery. This study aims to analyze consent practices and determine the proportion of patients being consented for bowel dysfunction.

Materials and methods: Consent forms for sphincter-preserving colorectal surgeries performed between August and November 2024 at a single center were analyzed for documentation regarding bowel dysfunction, and other complications were also noted.

Results: A total of 48 consent forms were analyzed, and we found that only 11/48 (22.9%) had documentation about postoperative bowel dysfunction being discussed with patients. We also found that postoperative pain was not documented in 19/48 (39.6%) cases, and anastomotic leaks were not reported in 8/48 (16.6%) cases. All patients (100%) consented to infections and bleeding; however, 3/48 (6.3%) did not consent to venous thromboembolism and damage to surrounding structures.

Conclusions: A low proportion of patients are counseled regarding postoperative bowel dysfunction at the time of consenting, and the issue should be discussed with all patients undergoing colorectal surgery as a part of standard practice.

## Introduction

Colorectal cancer is the third most common type of cancer in the United Kingdom, after breast cancer in females and prostate cancer in males. Surgery remains the mainstay of treatment with curative intent, aiming to excise the primary tumour (and associated lymph nodes), prevent recurrence and metastases, and improve quality of life [1]. There are a variety of procedures performed based on the location of the tumour, broadly divided anatomically into involving the right or left colon, sigmoid colon, rectum and anus. These procedures can be performed open, minimally invasive, or robot-assisted. As with any surgical procedure, there are numerous risks associated with surgery for colorectal cancer. It is essential to ensure that patients are adequately counseled about these risks and are provided with sufficient information to enable them to make an informed decision.

Consent is a core component of both medical and surgical practice, with increasing importance being given to discussions around consent, particularly after the Montgomery versus Lanarkshire Health Board case (2015). Attitudes toward consent have undergone a significant shift from a more paternalistic and authoritative approach to one of shared decision-making. Doctors are expected to provide patients with information regarding their diagnosis and prognosis, details about the procedure, the intended benefits, possible complications, alternative treatment options, and the likelihood of success. After this, the final decision rests with the patient. These criteria have been elaborated in the Royal College of Surgeons of England's ten steps of consent guidelines, as well as guidelines by the GMC [2]. Despite this, practices around consent vary significantly between centers, specialties, and surgeons. A study conducted in 2018 (after the Montgomery-Lanarkshire ruling) found that on the day of elective surgery, benefits, risks, and alternatives were discussed in less than half of the cases, while a copy of the documented discussion was provided to only 4.2% of patients [3].

Several potential complications associated with colorectal surgery should be discussed with patients. Pain (acute or chronic), infections (relating to the surgical site, septicemia, urinary/respiratory tract infections, etc.), bleeding (intraoperative or postoperative, possibly needing blood transfusions), damage to surrounding structures (bowel, bladder, neurovascular injury, etc.), and venous thromboembolism (deep vein thrombosis (DVT) or pulmonary embolism (PE)) are significant complications associated with most abdominal surgical procedures. Complications specific to colorectal surgery are intra-abdominal collections, anastomotic leaks, tumor recurrence, ileus or bowel obstruction, stoma-related complications, hernia formation, and long-term sexual or bowel dysfunction. Mortality varies based on the characteristics of the disease, the complexity and urgency of the procedure, and the patient's comorbidities. General anesthesia, although relatively safe, is also associated with certain risks that patients should be informed about.

A particular complication of colorectal surgery that is increasingly being discussed is the presence of bowel dysfunction postoperatively. This has been particularly noticed after anterior resections and is termed anterior resection syndrome (ARS) or low anterior resection syndrome (LARS). ARS is a collection of symptoms including (a) multiple or frequent passage of stools, (b) incomplete evacuation leading to clustering of bowel movements, (c) continence disorders ranging from flatus incontinence to minimal/major soiling, and (d) the sensation of urgency. The LARS score was developed in 2012, incorporating these symptoms to help aid the diagnosis. Although ARS has a variable course, symptoms usually peak in severity three to four months post surgery and persist up to one to two years later. Options for management are relatively limited and include dietary and lifestyle changes, anti-motility agents, retrograde colonic irrigation, nerve blocks, and pelvic floor rehabilitation, with stoma formation as the definitive last resort [4].

A meta-analysis conducted by Ye et al. in 2022 analyzed over 5,000 patients and concluded that risk factors for ARS postoperatively include a low height of the tumor and anastomosis, perioperative chemotherapy or radiotherapy, anastomotic leaks, and stoma dysfunction, while age and sex do not have a statistically significant influence [5]. A meta-analysis conducted by Croese et al. in 2018 found that the overall prevalence of persistent, significant LARS one year postoperatively was 41%, with reports ranging from 17.8% to 56%. The study also correlated with the meta-analysis by Ye et al., which found that anastomotic leaks, radiotherapy, and tumor/anastomotic height were associated with an increase in the prevalence of LARS [6].

Although not formally described as ARS, variable degrees of bowel dysfunction are common after any colorectal surgery, not just anterior resections. It is recognized particularly after reversal of stomas and is more commonly seen after procedures involving the left colon, especially sphincter-preserving surgeries. Any such procedure is vulnerable to anastomotic leaks, which increases the risk of longer-term disturbance in bowel function. Anastomotic leaks are associated with a twofold increase in requiring continence aids for fecal incontinence up to two years postoperatively. Another aspect is the creation of a defunctioning ileostomy, closure of which is also associated with bowel dysfunction due to various reasons; patients are more likely to have received radiotherapy, have lower rectal anastomoses, and have diversion colitis [7].

Any degree of long-term bowel dysfunction undoubtedly has a negative impact on patients' postoperative recovery, quality of life, and psychosocial well-being. Patients with minor and major bowel dysfunction reported disruption in social and sexual activities, with major bowel dysfunction also impacting sleep [8]. The impact on daily life can be illustrated with patients reporting having to implement dietary changes, restricting meal portions, being unable to exercise or pursue hobbies involving strenuous activity, having to travel with a change of clothes, social withdrawal, and dealing with stigma socially and at work [9].

It is therefore essential that patients are informed about ARS/bowel dysfunction at the time of consenting, which is not done in all cases. This study was undertaken to determine the prevalence of patients being provided with this information preoperatively for elective sphincter-preserving colorectal surgeries at a district general hospital, with the aim of surveying consent practices and implementing quality improvement.

## Materials and methods

This is a single-center study conducted at New Cross Hospital, Wolverhampton, United Kingdom. A retrospective search of operating theater lists of consultant colorectal surgeons was performed via the trust's internal database. We then identified patients undergoing sphincter-preserving colorectal surgeries over a four-month period from August to November 2024. Any procedure involving the creation of a stoma was excluded. Following this, preoperative consent forms for all patients were reviewed using electronic admission records via Clinical Web Portal, and details of postoperative complications documented at the time of consenting were noted. We initially identified 50 patients, of whom two were excluded from the final analysis: one due to having a consent form 4 (signed by a next of kin due to a lack of capacity) and one due to being unable to locate the consent form within the admission records. Final data collection involved 48 patients, and all documented complications were noted. These were then categorized into major (pain, infection, bleeding, venous thromboembolism, and damage to surrounding viscera) and minor (specific to colorectal procedures) complications for ease of interpretation. Postoperative bowel dysfunction, being the main focus of this study, was included with significant complications.

## Results

A total of 50 patients were identified, and 48 were included in the final analysis. One patient was excluded because their consent form was unavailable electronically, and another patient was excluded due to a lack of capacity to consent and the use of a different consent form, which a relative signed.

There were 28 male patients (58%) and 20 female patients (42%), with a mean age of 66.5 years. The mean length of stay was 9.6 days. All procedures were elective.

**Figure 1:**
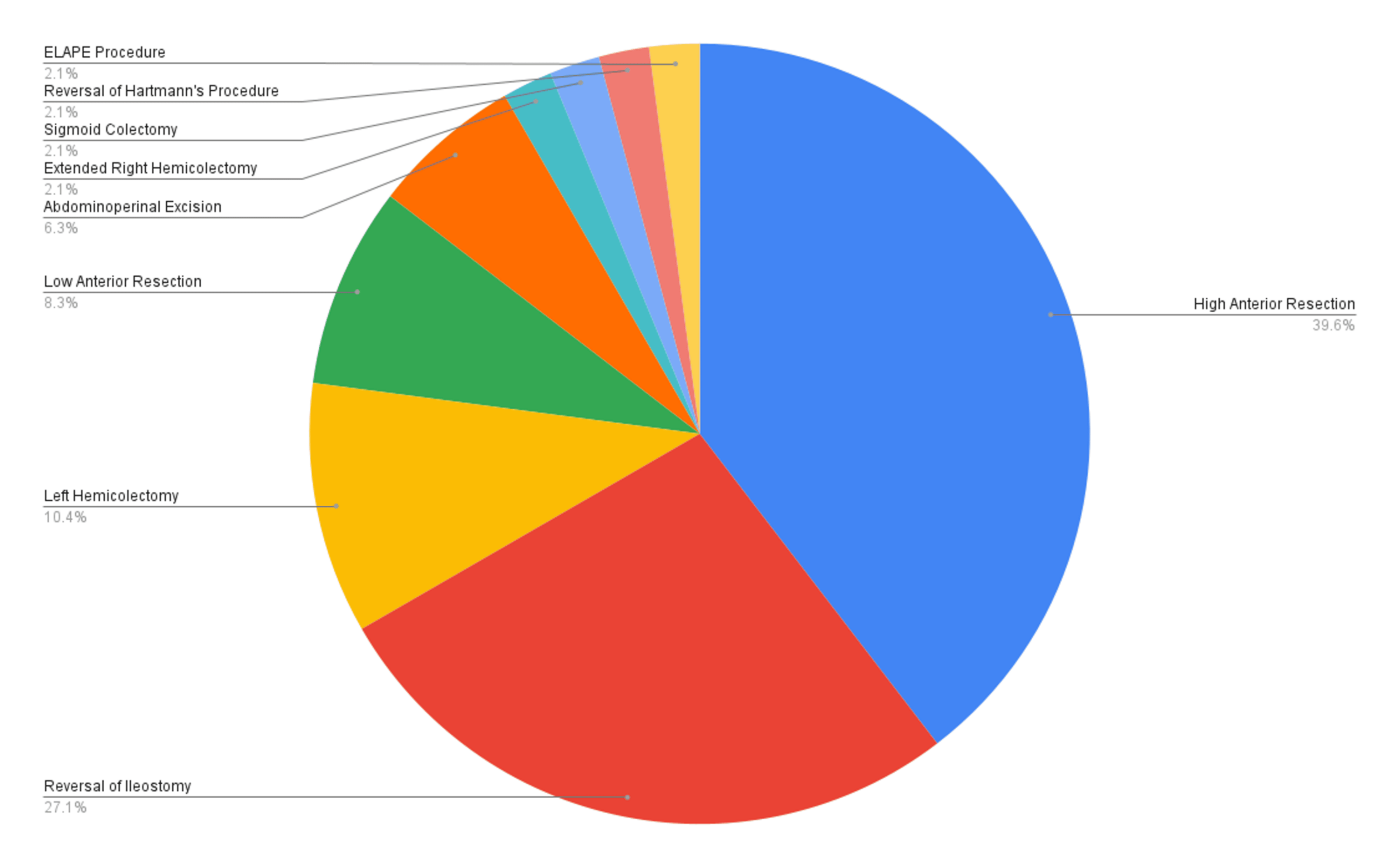
Details of surgeries performed ELAPE: extralevator abdominoperineal excision

The surgeries performed were 19 high anterior resections (39.6%), 13 reversals of ileostomies (27.1%), five left hemicolectomies (10.4%), four low anterior resections (8.3%), three abdominoperineal resections (6.3%), one extended right hemicolectomy (2.1%), one sigmoid colectomy (2.1%), one reversal of Hartmann's procedure (2.1%), and one extralevator abdominoperineal excision (ELAPE) procedure (2.1%). This is illustrated in Figure 1.

Forty-five of the 48 surgeries (93.8%) were performed due to colorectal cancer, two due to severe diverticular disease, and one due to severe endometriosis involving the bowel. Postoperative complications were classified into general major surgical complications (pain, infection, bleeding, damage to surrounding structures, and venous thromboembolism) and specific complications for ease of interpretation. Bowel dysfunction, being the main focus of this study, was included with general complications.

**Figure 2:**
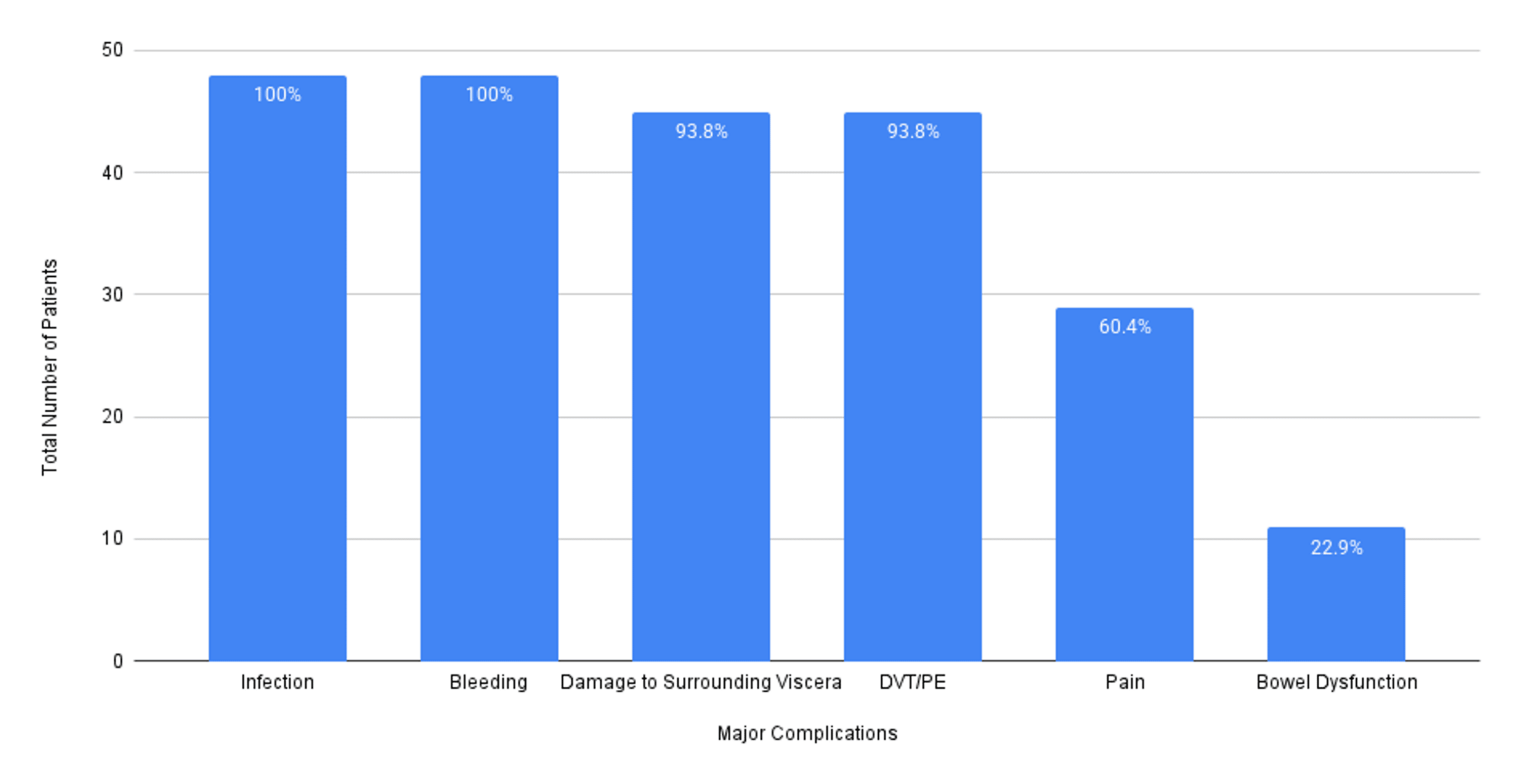
Proportion of patients consented for major surgical complications DVT: deep vein thrombosis, PE: pulmonary embolism

At the time of consenting, 48/48 patients (100%) were counseled regarding the risk of infections and bleeding; 45/48 patients (93.8%) were counseled regarding the risk of damage to surrounding structures and venous thromboembolism; 29/48 patients (60.4%) were counseled regarding postoperative pain; and 11/48 patients (22.9%) were counseled regarding the risk of postoperative bowel dysfunction. This is illustrated in Figure 2.

Phrasing varied significantly across consent forms. Some simply mentioned "infections" and "clots," while others elaborated on the types of infections (wound, urinary/respiratory, COVID-19, etc.) and clots (DVT/PE). Similarly, some mentioned "damage to surrounding viscera," while others specified the specific structures involved, such as the bladder, nerves, and blood vessels. Regarding bowel dysfunction, 5/11 (45%) cases mentioned "altered bowels," 2/11 (18%) cited "change in bowel function," and 4/11 (18%) cases mentioned "(low) anterior resection syndrome."

**Figure 3:**
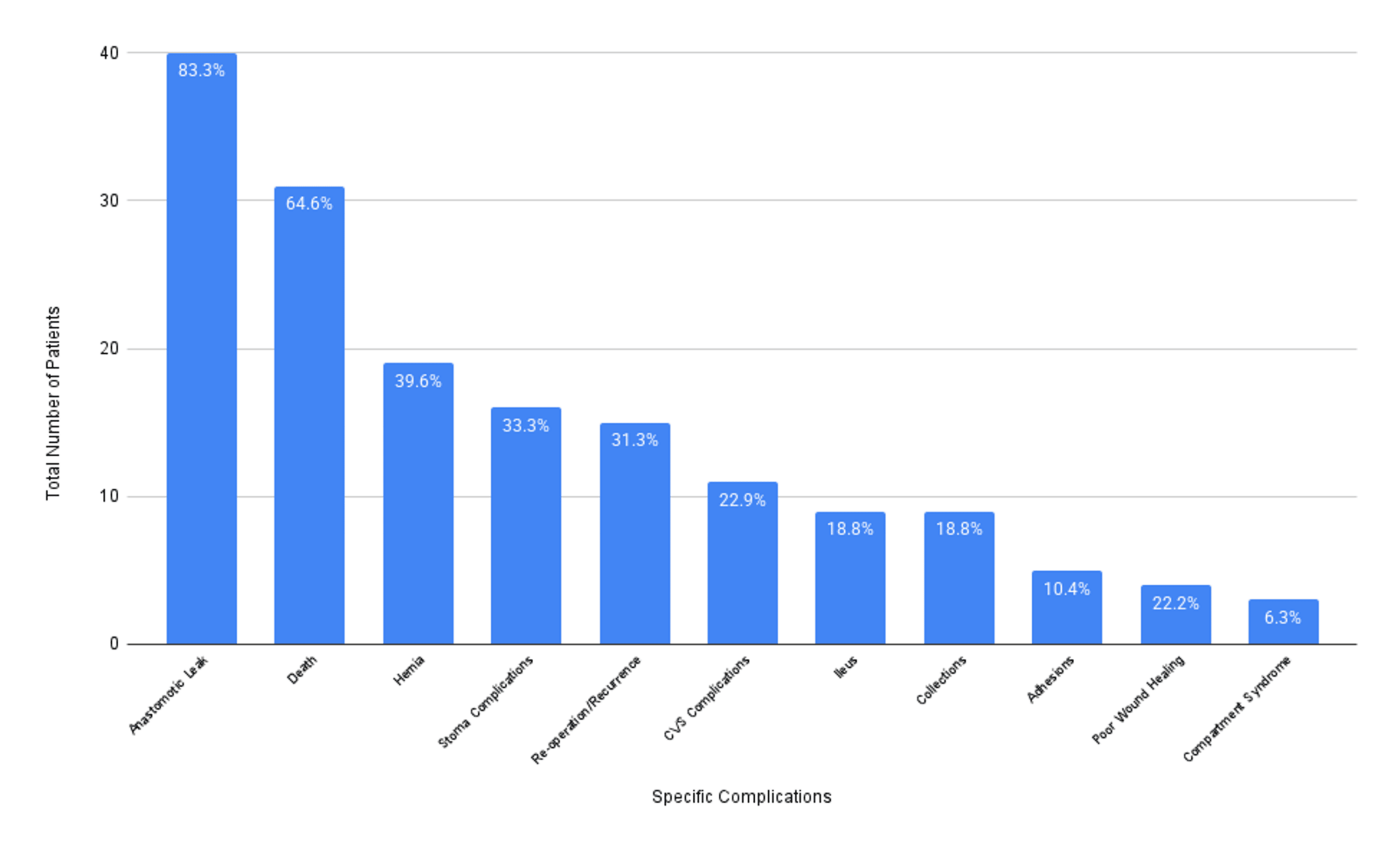
Proportion of patients consented for specific surgical complications CVS: cardiovascular

Regarding specific complications, the most frequently mentioned was anastomotic leak, with 40/48 patients (83%) being informed about the risk. Other complications mentioned in decreasing order of frequency were death, hernias, stoma-related complications, the need for reoperation, cardiovascular complications, ileus, collections, adhesions, poor wound healing, and compartment syndrome. This is illustrated in Figure 3.

## Discussion

This study highlighted several shortcomings in current consent practices. Despite the high incidence of postoperative bowel dysfunction as described above, only 23% of patients were informed about this preoperatively. Looking specifically at anterior resections, there were a total of 23 procedures performed (19 high + 4 low), out of which patients were informed about ARS/LARS in only four cases (17%). Also noteworthy is that in four out of 11 documentations about bowel dysfunction, the term "(low) anterior resection syndrome" had been written without further explanation on the consent form, which is of no meaning to the patient. In the other cases, "changes to bowel function" or "altered bowels" were documented, which is understandable for patients but still lacks sufficient detail. Ideally, a description of the type and duration of symptoms should be documented and discussed with patients, including the impact on quality of life.

Another interesting observation is that not all patients consented regarding postoperative pain, with this being documented in approximately 60% of cases. Even if it is assumed that patients know about and expect pain after surgery, they should, regardless, be informed as a matter of good clinical practice. Postoperative pain can be acute and resolve in a short time period, but in some cases, it can persist as chronic pain, which can be very distressing for individuals. Chronic post-surgical pain has been found to affect up to 11.8% of patients 12 months postoperatively, with 2.2% reporting severe pain [10]. This should be part of the discussion when gaining informed consent.

In terms of other significant complications, it was encouraging to note that all patients were informed about the risks of bleeding and infections, and most were also informed about the risks of damage to surrounding viscera and venous thromboembolism. In terms of specific complications, however, the results were variable. Anastomotic leaks and ileus are two of the most common complications after colorectal surgery, often prolonging the length of stay and complicating recovery. Despite advances in surgical techniques, the incidence of anastomotic leaks is reported to be as high as 30%, thus being a significant source of morbidity (20-35%) and mortality (2-16.4%) [11]. In terms of ileus, a systematic review analyzed almost 30,000 patients and reported an incidence of 9.5% for postoperative ileus after colorectal surgery, with some studies reporting an incidence of up to 30% [12]. In this study, 83% of patients consented to discussing anastomotic leaks; however, only 18.8% consented to discussing ileus. More patients consented to death than to ileus, despite the latter being much more common.

It was interesting to note that complications like bleeding, infections, venous thromboembolism, damage to visceral structures, and anastomotic leaks, which have a higher chance of medicolegal consequences for the surgeon, were mentioned in a high proportion of cases. In contrast, bowel dysfunction, pain, and ileus, which are generally perceived to be less serious than the above, were not discussed sufficiently. It is essential to remember the impact these complications have on patients' quality of life, which can be permanent. Therefore, it should be a standard part of practice to counsel patients regarding these risks. Lastly, it was also interesting to note that not a single consent form mentioned the risks associated with general anesthesia. While primarily the responsibility of the anesthesiologist, it is often the case that patients are reviewed and consented to by a surgeon before meeting an anesthesiologist. The question remains debatable as to whether surgeons should inform patients about these risks.

Limitations and future potential

This is a retrospective study conducted at a single district general hospital over a limited period aimed at improving local quality. The focus was placed on sphincter-preserving colorectal procedures, and the sample size is relatively small. The study aimed to determine the proportion of patients who consented to postoperative bowel dysfunction; therefore, consent practices for all individual-specific complications were not analyzed in much detail. Despite these limitations, this study demonstrates a lack of informed consent regarding postoperative bowel dysfunction. Future potentials include conducting the same analysis over a longer time period, performing a second cycle of the study after local presentations to assess the impact, and reproducing this study at other centers locally and nationally to determine general consent practices.

## Conclusions

Bowel dysfunction or (low) anterior resection syndrome is a well-recognized complication affecting patients undergoing colonic or rectal surgery and significantly impacts postoperative quality of life. Despite this, only a low proportion of patients undergoing colorectal surgeries are counseled regarding this and provide informed consent. Providing adequate information and counseling in this regard at the time of consenting should form a standard part of practice.

## References

[1] Rentsch M, Schiergens T, Khandoga A, Werner J (2016). Surgery for colorectal cancer - trends, developments, and future perspectives. Visc Med.

[2] Sebastian A, Wyld L, Morgan JL (2024). Examining the variation in consent in general surgery. Ann R Coll Surg Engl.

[3] Knight SR, Pearson R, Kiely C, Lee G, MacDonald AJ, Macdonald A (2019). Patient consent in the post-Montgomery era: a national multi-speciality prospective study. Surgeon.

[4] Sarcher T, Dupont B, Alves A, Menahem B (2018). Anterior resection syndrome: what should we tell practitioners and patients in 2018?. J Visc Surg.

[5] Ye L, Huang M, Huang Y, Yu K, Wang X (2022). Risk factors of postoperative low anterior resection syndrome for colorectal cancer: a meta-analysis. Asian J Surg.

[6] Croese AD, Lonie JM, Trollope AF, Vangaveti VN, Ho YH (2018). A meta-analysis of the prevalence of low anterior resection syndrome and systematic review of risk factors. Int J Surg.

[7] Lam D, Jones O (2020). Changes to gastrointestinal function after surgery for colorectal cancer. Best Pract Res Clin Gastroenterol.

[8] Maalouf MF, Robitaille S, Penta R (2023). Understanding the impact of bowel dysfunction on quality of life after rectal cancer surgery from the patient’s perspective. Dis Colon Rectum.

[9] Maalouf MF, Wang A, Robitaille S, Liberman AS, Fiore JF Jr, Feldman LS, Lee L (2024). Patient perspective on adapting to bowel dysfunction after rectal cancer surgery. Colorectal Dis.

[10] Thapa P, Euasobhon P (2018). Chronic postsurgical pain: current evidence for prevention and management. Korean J Pain.

[11] Rennie O, Sharma M, Helwa N (2024). Colorectal anastomotic leakage: a narrative review of definitions, grading systems, and consequences of leaks. Front Surg.

[12] Emile SH, Horesh N, Garoufalia Z, Gefen R, Ray-Offor E, Wexner SD (2024). Strategies to reduce ileus after colorectal surgery: a qualitative umbrella review of the collective evidence. Surgery.

